# Subcutaneous panniculitis‐like T‐cell lymphoma in a young girl presenting with periorbital edema and fever: A case report

**DOI:** 10.1002/ccr3.5462

**Published:** 2022-02-13

**Authors:** Seyed Mohamad Kazem Nourbakhsh, Mohammad Bahadoram, Farid Kosari, Mehrdad Jafari, Nahid Aslani, Shakiba Hassanzadeh

**Affiliations:** ^1^ 48439 Department of Pediatric Hematology and Oncology Imam Khomeini Hospital Complex Tehran University of Medical Sciences Tehran Iran; ^2^ Thalassemia and Hemoglobinopathy Research Center Health Research Institute Ahvaz Jundishapur University of Medical Sciences Ahvaz Iran; ^3^ 48439 Department of Pathology School of Medicine Shariati Hospital Tehran University of Medical Sciences Tehran Iran; ^4^ Department of Otorhinolaryngology Imam Khomeini hospital complex Tehran University of Medical Sciences Tehran Iran; ^5^ Department of Pediatrics Tehran University of Medical Science Tehran Iran

**Keywords:** lymphoma, periorbital swelling, subcutaneous panniculitis‐like T‐cell lymphoma, systemic lupus erythematosus, T‐cell lymphoma

## Abstract

Subcutaneous panniculitis‐like T‐cell lymphoma is a rare and highly malignant extra‐nodal lymphoma. It has a wide range of clinical presentations (such as periorbital swelling as in our case) and should be considered in the differential diagnosis of systemic lupus erythematosus, especially in children.

## Graphical abstract

Subcutaneous panniculitis‐like T‐cell lymphoma is a rare and highly malignant extra‐nodal lymphoma. It has a wide‐range of clinical presentations (periorbital swelling as in our case) and should be considered in the differential diagnosis of systemic lupus erythematous, especially in children. Biopsy and pathological evaluation is essential for a definite diagnosis.

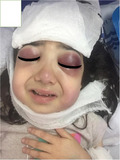

## 1 INTRODUCTION

Subcutaneous panniculitis‐like T‐cell lymphoma (SPTCL) is a rare and highly malignant extra‐nodal lymphoma that preferentially infiltrates into the subcutaneous adipose tissue. To our knowledge, there have been no reported cases of SPTCL with periorbital swelling. We report a very rare case of SPTCL complicated by severe periorbital swelling in a young girl.

Subcutaneous panniculitis‐like T‐cell lymphoma (SPTCL) is a peripheral T‐cell lymphoma derived from a mature cytotoxic T cell. SPTCL accounts for less than 1% of non‐Hodgkin lymphomas (NHLs).^1^ The exact incidence of SPTCL is unknown. It is most commonly seen in the fourth to sixth decades of life with a median age of 36 years. There is a female predominance and about 20% of the patients have an associated autoimmune disease such as systemic lupus erythematosus (SLE). Patients with SPTCL typically present with one or more subcutaneous nodules (usually painless) or poorly circumscribed indurated plaques that involve the extremities or trunk.^2^, ^3^ SPTCL is diagnosed with a deep skin biopsy that includes the sub‐cutis. SPTCL is characterized by subcutaneous infiltration of atypical lymphocytes that express CD3, CD8, alpha/beta T‐cell receptors, and cytotoxic granule proteins.^2^, ^4^ We herein report a very rare case of SPTCL in a child that presented with a facial lesion and high‐grade fever.

## 2 CASE REPORT

**FIGURE 1:**
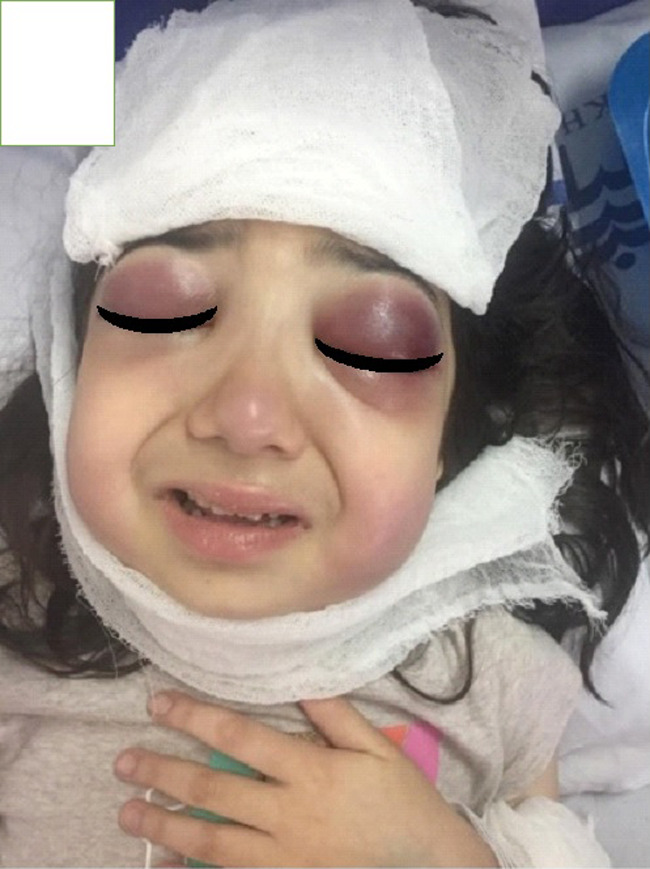
Edema of the face, mostly in the periorbital area

A previously healthy ten‐year‐old girl was referred to our hospital due to a right periorbital swelling, weakness, and high‐grade fever. She had developed the swelling about one week before admission (Figure 1) and had been empirically treated with an antibiotic with a possible diagnosis of pre‐septal cellulitis. However, the lesion had spread to the cheeks and had become ulcerative. There was a history of SLE in the patient's grandmother and aunt.

**FIGURE 2:**
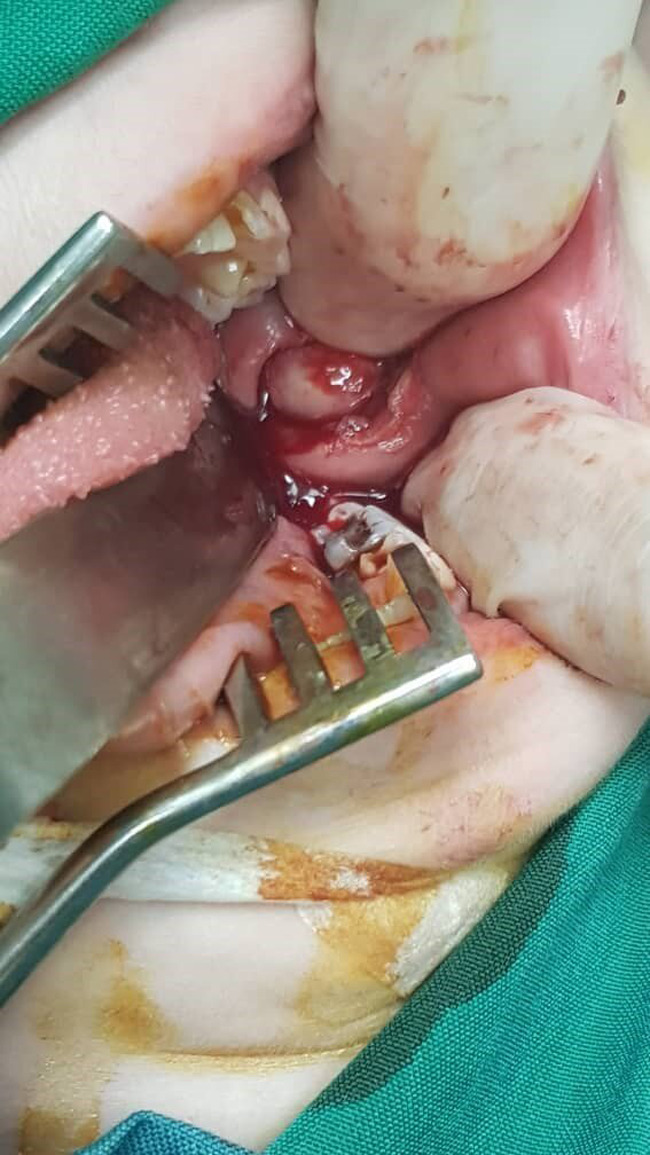
Image of the excisional buccal biopsy performed by an otorhinolaryngologist

**FIGURE 3:**
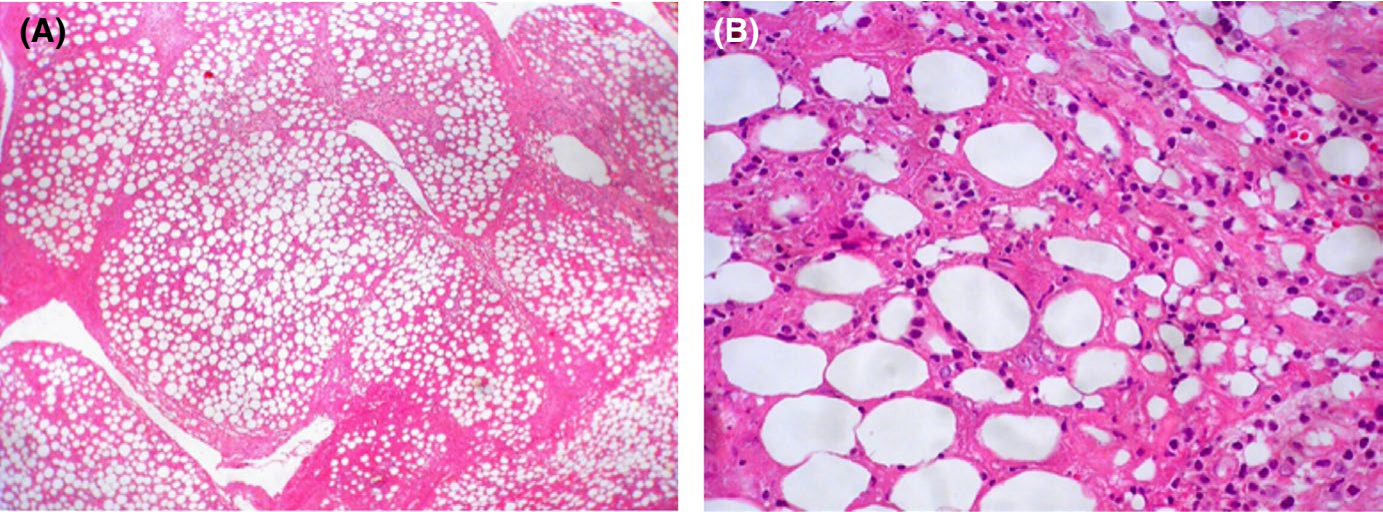
(A) Low‐power view of the tumor showing excessive infiltration of the subcutaneous fat (H&E×40). (B) High‐power view of the tumor showing excessive lymphocytic infiltration and rimming of the fat cells with atypical lymphoid cells (H&E×400)

**FIGURE 4:**
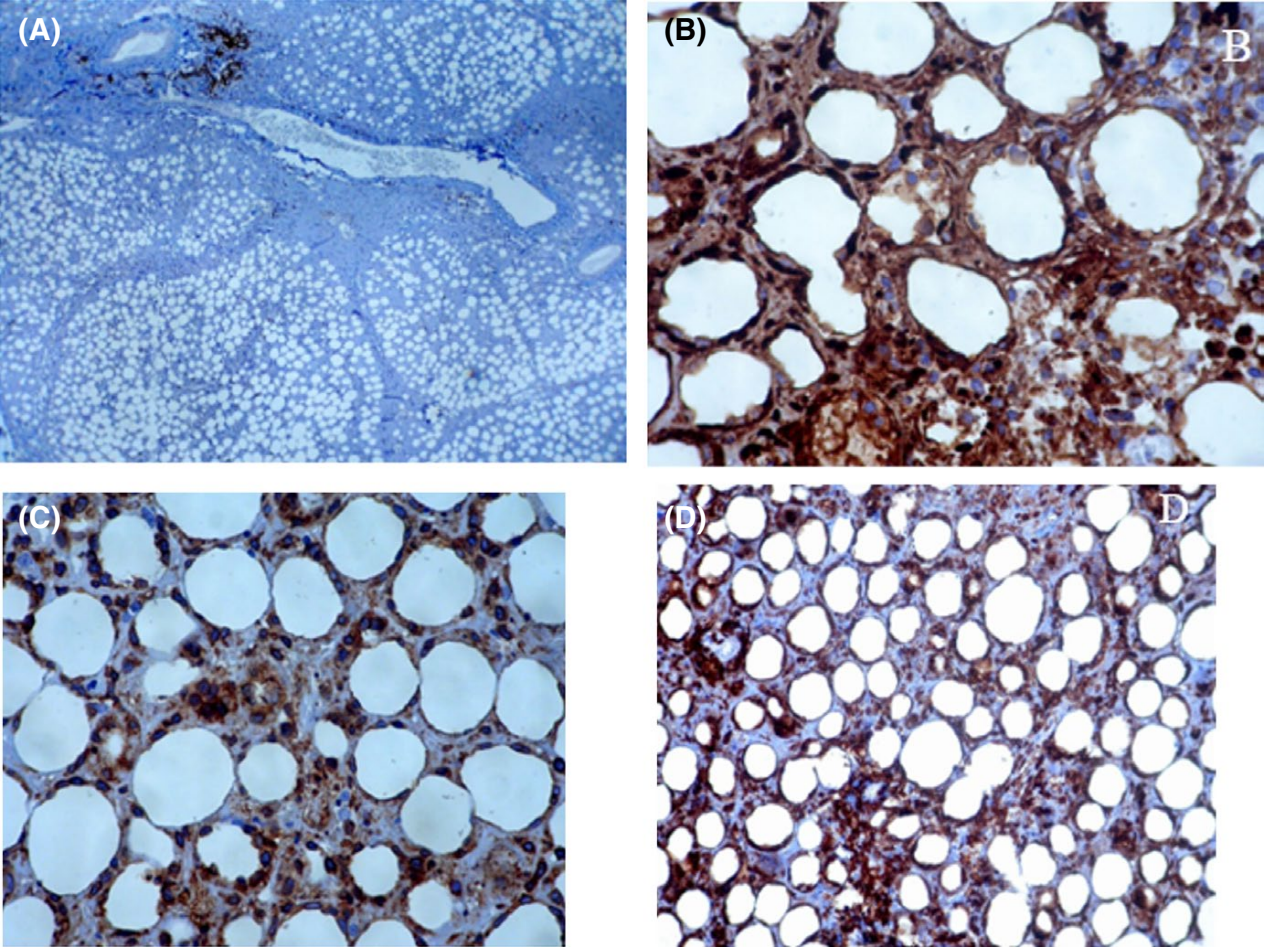
Immunohistochemical staining of the tumor cells showed negative staining for CD20 (A), and positive CD3 (B), CD8 (C), and granzyme B (D). There was typical peripheral rimming of the adipocytes with neoplastic cytotoxic T lymphocytes

On evaluation she had leukopenia, lymphopenia, and mildly elevated inflammatory markers. Ultrasound of the lesion showed extensive edema of the soft tissue in the face and upper neck, particularly in the superficial subcutaneous tissue. Magnetic resonance imaging (MRI) revealed cellulitis without any fluid collection or lymphadenitis in the subcutaneous area of the bilateral cheek and submandibular regions. Evaluation with metaiodobenzylguanidine (MIBG) scintigraphy was negative. Bone marrow evaluation was normal, and there was no evidence of inflammatory involvement. Finally, the patient underwent an excisional buccal biopsy (Figure 2). The results of the pathological evaluation and immunohistochemistry (IHC) examination suggested panniculitis, necrotizing leukocytoclastic vasculitis, and lobular panniculitis. The results of the second pathological evaluation revealed SPTCL as the diagnosis (Figures 3 and 4). The details of the patient's pathological evaluations and IHC results are shown in Table 1. Given the patient's systemic illness, persistent fever, no response to broad‐spectrum antibiotics, and negative cultures and infectious evaluations, empiric treatment with methylprednisolone was initiated for three consecutive days. It was temporarily discontinued for several days. However, her clinical condition did not improve. Laboratory results were positive for fluorescent antinuclear antibody (FANA) with a titer of 1/320 and angiotensin‐converting enzyme (ACE) of 110 U/L but were normal for other autoantibodies and complements. Another biopsy was performed and the results showed a diagnosis of panniculitis‐like T‐cell lymphoma (Table 1), and the patient was planned to undergo chemotherapy. However, at the initiation of chemotherapy, she developed severe fever, pancytopenia, high serum ferritin, and an increased level of triglyceride. Consequently, a repeat bone marrow examination was performed which revealed hemophagocytic cells. Therefore, hemophagocytic syndrome was diagnosed and the patient was treated with methylprednisolone, intravenous immunoglobulins (IVIGs), and cyclosporine. The patient's clinical features and laboratory results improved after two weeks of treatment. Consequently, chemotherapy was initiated. Following chemotherapy, her fever was discontinued and the facial swelling decreased significantly. The patient was discharged and on the follow‐up visit, she was afebrile and the swelling and ulcerative facial lesions had completely improved.

**TABLE 1:** Details of the patient's pathological evaluations

Pathology Evaluation	Pathology Report	Diagnosis
First evaluation	An ovoid encapsulated elastic mass: Size: 1.8 x 1 x 1.1 cm Microscopic pathologic evaluation: Uniform adipocytes with intervening thin fibrous bands and lobules of fatty tissue with fibroid necrosis Thrombosis in small vessels The large vessels were surrounded by some atypical lymphoid cells that had small hyperchromatic nuclei with irregular nuclear contours and scanty cytoplasm. Peripheral rimming of adipocytes with atypical lymphoid cells. Evaluation with IHC: Most lymphoid cells: Positive: ▪ CD3, CD7, CD19, CD20 Significantly positive: ▪ CD8 and granzyme B Negative: ▪ CD4, CD34, CD56, c‐kit, TdT, MPO	Panniculitis Necrotizing leukocytoclastic vasculitis Lobular panniculitis.
Second evaluation	The histopathologic features (Figure 3): Diffuse infiltration of the subcutaneous fat. Presence of isolated and aggregates of small to medium lymphocytes with hyperchromatic nuclei, irregular nuclear contours, and scanty pale‐stained cytoplasm. Presence of many apoptotic bodies. Presence of typical rimming of the adipocytes with infiltrating atypical lymphoid cells IHC staining: Positive: CD3, CD8, granzyme B (Figure 4) Negative: CD20, CD4, CD123	SPTCL
Third evaluation	Severe infiltration of CD8+ lymphocyte cells in the subcutaneous area without immunological complex deposition.	Panniculitis‐like T‐cell lymphoma

Abbreviations: Immunohistochemistry (IHC), myeloperoxidase (MPO), terminal deoxynucleotidyl transferase (TdT), and subcutaneous panniculitis‐like T‐cell lymphoma (SPTCL).

## 3 DISCUSSION

Panniculitis is the inflammation of the subcutaneous fat. Histopathologically, panniculitis mostly affects the lobules or septa regardless of vascular involvement.^5^ A patchy lymphoplasmacytic infiltrate in the lobules of the subcutaneous fat along with lymphocytic nuclear dust is a clue for the histopathological diagnosis of lupus erythematosus (LE) panniculitis (LEP).^6^ The peripheral T‐cell lymphomas (PTCLs) are a heterogeneous group of generally aggressive neoplasms that constitute less than 1% of all NHLs in adults. SPTCL is a type of PTCL that is derived from a mature cytotoxic T cell that commonly mimics panniculitis.^7^ The patients present with subcutaneous nodules or plaques that, on pathologic evaluation, demonstrate cellular infiltrates in the subcutaneous fat, usually sparing the overlying epidermis. Patients with SPTCL typically present with one or more subcutaneous nodules that are usually painless or poorly circumscribed indurated plaques. The most commonly involved areas include the legs (71%), arms (62%), trunk (56%), and/or face (25%).^3^, ^8^ The diameter of the nodules can vary from 0.5 cm to more than 20 cm. Necrosis may be present but ulceration is uncommon (6%). Single lesions are rare and approximately 80% of the patients have multiple nodules and/or plaques. In addition, the lesions may have been previously misdiagnosed as panniculitis or other cutaneous diseases resulting in median times from lesion development to the diagnosis of seven months. Systemic B symptoms and bone marrow abnormalities are reported in approximately 60% and 20–30% of the patients, respectively.^3^, ^8^, ^9^ This case presented with right periorbital swelling, weakness, and high‐grade fever. Bone marrow involvement by lymphoma is highly unusual in these patients. The most common bone marrow abnormality is hemophagocytosis and about 17% of the patients develop secondary hemophagocytic lymphohistiocytosis (HLH or hemophagocytic syndrome).^3^, ^8^ The present case was also diagnosed with hemophagocytic syndrome and treated with methylprednisolone, IVIGs, and cyclosporine. Approximately, 45% of the patients with SPTCL have laboratory abnormalities (anemia, leukopenia, thrombocytopenia, and/or elevated liver function tests). This is in line with our case that had leukopenia, lymphopenia, and mildly elevated inflammatory markers. Radiologic abnormalities on computed tomography (CT) scans (such as lymphadenopathy, hepatomegaly, and pleural effusions) are seen in less than 10% of the patients. Skin biopsy demonstrates a subcutaneous infiltrate of atypical lymphocytes involving the fat lobules but typically spares the septate, overlying dermis, and epidermis (Figure 3). The neoplastic cells rim the individual fat cells and may invade the deeper dermis, surrounding the sweat glands and/or hair follicles.^4^, ^10^ Fat necrosis and karyorrhexis are common, and vascular invasion is seen in some cases. The diagnosis of SPTCL is made with a deep skin biopsy that includes the subcutaneous tissue (such as an excisional biopsy). The diagnosis is based on the pathology and immunophenotypic findings described above along with characteristic clinical features. Repeat biopsies may be required. However, shave biopsies are not appropriate since they do not include subcutaneous tissue. Benign and malignant lesions that have lymphocyte infiltration of the subcutaneous fat are the differential diagnosis of SPTCL.^11^ For example, SPTCL may resemble panniculitis. However, in contrast to SPTCL, benign panniculitis does not have cytologic atypia or rearrangements of the clonal T‐cell receptor gene. Additionally, LE presents more on the face and proximal extremities. SPTCL usually presents on the lower extremities, upper extremities, and trunk. Fever and hepatosplenomegaly are more likely seen in SPTCL.^12^ However, they can also be observed in LE. The diagnosis of LEP may be challenging in cases where the involvement of subcutaneous fat is the only manifestation of the disease. Clinically, LEP and subcutaneous SPTCL are indistinguishable, especially if there is epidermis involvement. In SPTCL there are atypical CD8+ T‑lymphocytes and no septal fibrosis, B‑cell follicles, and plasma cells. However, LEP usually has an interface change, a mixture of CD4 and CD8 T cells, lymphoid follicles or B‐cell aggregates, aggregates of CD123+ plasmacytoid dendritic cells, plasma cell aggregates, and intradermal mucin. Other helpful findings in distinguishing SPTCL from LEP are a low Ki67 proliferation index in the T cells that rim the adipocytes and no presence of T cells that are cytologically atypical.^3^, ^13^, ^14^ In addition, inflammatory cells such as eosinophils, neutrophils, and plasma cells are mostly seen in LE.^8^ A combination of the clinical features, histology, immunophenotyping, molecular analysis, and possibly repeat biopsies may be required to differentiate SPTCL from LEP. However, some cases remain ambiguous.^15^ For example, lupus profundus panniculitis presents as a firm nodular lesion in patients with SLE and these nodules are usually painful and can appear in the mid‐dermal, deep dermal, or subcutaneous layers (in contrast to SPTCL).^16^ Histological examination shows perivascular infiltrates of mononuclear cells plus panniculitis which present as hyaline fat necrosis with mononuclear cell infiltration and lymphocytic vasculitis (Figure 3 A‐B). Furthermore, unlike SPTCL, lupus profundus panniculitis does not have rearrangement clonality on the T‐cell receptor gene.

Most patients with PTCL that undergo treatment will either not achieve remission or experience relapse.^17^ Therefore, these patients undergo a second‐line combination chemotherapy. Those patients that respond to these therapies should be offered to have autologous or allogeneic hematopoietic cell transplantation because of the potential of long‐term disease‐free survival after these procedures. There is no long‐term response after chemotherapy alone. There have been reports of SPTCL. For example, Kini et al. described two male patients with multiple subcutaneous nodules. One of these patients received a cycle of CHOP (cyclophosphamide, vincristine, doxorubicin, and prednisolone) followed by systemic steroids.^5^ Dong et al. have also reported a relapsed case of SPTCL in a 15‐year‐old woman which responded effectively to chemotherapy.^18^ Furthermore, Hrudka et al. reported a case of a 65‐year‐old man with SPTCL and severe hemophagocytic lymphohistiocytosis.^9^ Another study reported a 33‐year‐old woman with SPTCL in her right upper eyelid which was treated with prednisone, methotrexate, and interferon‐α.^19^ Our patient also showed good response to treatment with methylprednisolone, IVIGs, cyclosporine, and chemotherapy.

## 4 CONCLUSION

We report a young girl who presented with fever, facial swelling, and painless ulcerative lesions as well as a hemophagocytic syndrome. She was diagnosed with SPTCL and showed good response to chemotherapy.

## CONFLICT OF INTEREST

The authors declare no conflict of interests.

## AUTHOR CONTRIBUTION

All authors passed four criteria for authorship contribution based on recommendations of the International Committee of Medical Journal Editors. All authors had equal contribution to this study. All authors have read and approved the final manuscript.

## ETHICAL APPROVAL

This study was conducted following the principles of the World Medical Association Declaration of Helsinki.

## CONSENT

Written informed consent was obtained from the patient's parents for the publication of this case report and any accompanying images.

## Data Availability

Data sharing is not applicable—no new data generated.
